# Neurological Manifestations Related to Immune Checkpoint Inhibitors: Reverse Translational Research by Using the European Real-World Safety Data

**DOI:** 10.3389/fonc.2022.824511

**Published:** 2022-03-15

**Authors:** Rosanna Ruggiero, Barbara Stelitano, Federica Fraenza, Gabriella di Mauro, Cristina Scavone, Liberata Sportiello, Concetta Rafaniello, Raffaella Di Napoli, Romano Danesi, Marzia Del Re, Francesco Rossi, Annalisa Capuano

**Affiliations:** ^1^ Campania Regional Centre for Pharmacovigilance and Pharmacoepidemiology, Naples, Italy; ^2^ Department of Experimental Medicine – Section of Pharmacology “L. Donatelli”, University of Campania “Luigi Vanvitelli”, Naples, Italy; ^3^ Unit of Clinical Pharmacology and Pharmacogenetics, Department of Clinical and Experimental Medicine, University Hospital of Pisa, Pisa, Italy

**Keywords:** immune checkpoint inhibitors, neurological toxicity, immune-related adverse events, immunotherapy, post-marketing surveillance, translational research, EudraVigilance database

## Abstract

Immune checkpoint inhibitors (ICIs) are widely used improving clinical outcomes in many cancer patients. However, they can induce serious consequences, like neurological immune-related adverse drug reactions (NirADRs). Although these are rare complications, they can be serious with important impact on patients’ quality of life. Our purpose is to describe these adverse events observed in the European clinical practice context. We carried out a descriptive analysis of individual case safety reports (ICSRs) related to ICIs collected until February 7, 2020, in the European spontaneous reporting database, EudraVigilance, and reported nervous disorders as suspect adverse drug reactions (ADRs). NirADRs were classified according to the Medical Dictionary for Regulatory Activities (MedDRA). In order to identify a hypothetical different reporting probability of the NirADR types between the ICI classes, we carried out a disproportionality analysis. The reporting odds ratio (ROR) with 95% CI was computed comparing the different ICI classes to each other based on their pharmacological target [the cytotoxic T-lymphocyte antigen-4 (CTLA-4), the programmed death-1 (PD-1) or its ligand (PD-L1)]. Finally, we researched in the literature the hypothesized mechanisms, which could explain the onset of these ICI-related neurological complications. Overall, we found 4,875 cases describing 6,429 ICI-related suspected NirADRs. ICI-related neurotoxicities include a wide range of central and peripheral events. These were mainly related to anti-PD-1 agents and occurred in male patients (59%). Our analysis confirmed a gender difference of NirADRs. Twenty-three percent of the events (comprising myasthenia gravis, neuropathy peripheral, and cerebral infarction) had unfavorable fallouts, including fatal outcome (7%). Majority of the NirADRs were categorized as “Neurological disorders NEC” HLGTs MedDRA (2,076; 32%). In 1,094 cases (22%), more NirADRs overlapped with other neurologic complications. An interesting overlapping of myasthenia gravis with myositis or myocarditis emerged. From our disproportionality analysis, an increased reporting probability of peripheral neuropathies and headaches emerged with ipilimumab when compared to anti-PD-1 and anti-PD-L1 agents. However, neuromuscular disorders were more probably reported with anti-PD-1. Several pathogenic mechanisms, including neuronal damage by T cells and autoantibodies and/or cytokine-mediated inflammation processes, have been hypothesized. However, the pathogenesis of these ICI-related complications is not completely understood. Considering the recent marketing authorizations of ICIs, further studies are strongly needed to monitor their neurologic safety profile.

## 1 Introduction

Immune checkpoint blockade therapies have significantly improved the prognosis and survival of cancer patients, representing an important and innovative milestone in the field of immuno-oncology (1, 2). The available immune checkpoint inhibitors (ICIs) are monoclonal antibodies targeting the cytotoxic T-lymphocyte antigen-4 (CTLA-4), the programmed death-1 (PD-1), or its ligand (PD-L1) (3). Other novel immune checkpoint inhibitors, such as the lymphocyte-activation gene 3 (LAG-3), the T-cell membrane protein-3 (TIM-3), and the T-cell immunoglobulin and ITIM domain (TIGIT), are under research as drug targets (4). By inhibiting the interaction between the immune checkpoints expressed on T cells, such as PD-1 and CTLA-4 and their ligands (PD-L1 and CD80/86) expressed by tumor cells/antigen-presenting cells, ICIs induce the activation of T effector cells targeting tumor cells (5). To date, in Europe, seven ICIs have been authorized by the European Medicines Agency (EMA), namely, an anti-CTLA-4 agent (ipilimumab, the first one authorized in 2011), three PD-1 inhibitors (pembrolizumab and nivolumab, both authorized in 2015, and cemiplimab, authorized in June 2019), and three anti-PD-L1 agents (atezolizumab, avelumab, and durvalumab, authorized between 2017 and 2018). Since 2015, the combination therapy with nivolumab and ipilimumab was also approved by the EMA, allowing to obtain long-term responses in higher percentages of patients (up to 60%) compared with monotherapies (20%–40%) (6). Other additional ICIs have already been authorized in China and the USA, like tremelimumab and camrelizumab (7). New generations of ICIs in mono- or combination therapies are actually undergoing preclinical and clinical development in order to obtain safer and more effective cancer treatments (8). According to a recent review published in 2019 on the Nature journal, ICIs are the first therapeutic class active in the field of immuno-oncology (9, 10). Given their ability to induce long-term durable responses, these innovative drugs are becoming the standard of care for multiple solid tumor types, including non-small-cell lung cancer, melanoma, renal cell cancer, and carcinoma of the head and neck (11). Unfortunately, the major difficulty in the application of ICIs is immunotherapy resistance that an important proportion of patients experience (12). In fact, not all patients would significantly respond to ICI treatments. Differences on a patient-to-patient basis emerged in the obtained outcomes (13). So, in a precision medicine view, the identification of the most effective biomarkers to predict ICI response, to select the patients, and to maximize the immunotherapy benefit remains an important challenge (14, 15).

Although several clinical trials have shown their efficacy, in addition to resistance gaps, some specific safety concerns remain to be monitored (16). Since immune checkpoints are critical in maintaining immunologic homeostasis, their inhibition can induce new complex immune-mediated adverse effects, commonly known as immune-related adverse events (irAEs), which are not induced by conventional cytotoxic anticancer agents (17). More commonly, when they occurred, irAE complications involve cutaneous, gastrointestinal, or endocrine systems (18). However, these treatments can also induce rare but clinically serious and potentially life-threatening consequences, such as neurological or cardiac adverse events (1, 19). Neurological outcomes were infrequently reported in pre-marketing studies. However, these complications continue to be described by clinicians and patients in the post-marketing experience (20). ICI-related neurotoxicity can involve the central and the peripheral nervous system. Approximately 1%–6% of patients receiving ICIs develop serious neurological outcomes (21).

Several studies were conducted in order to observe these unwanted events in clinical practice context. Recently, Johnson et al. investigated the neurological safety profiles of ICIs evaluating data from the international surveillance database VigiBase. According to their results, the ICI administration was associated with overreporting of specific neurological complications such as myasthenia gravis, encephalitis, non-infectious meningitis, cerebral vasculitis, and Guillain–Barré syndrome. Although it rarely occurs, the overlapping with other neurological or some non-neurological complications has been reported, influencing the fatality rate of particular events (22). In light of this, prompt diagnosis and adequate treatment of these complications (even though uncommon) are needed to reduce the risk of their long-term morbidity or mortality (11, 21). It is necessary to keep high the clinician’s attention for their correct identification as well as to identify the potential mechanisms underlying these complications in order to make their management more efficient and adequate.

Although some studies have already investigated the onset of specific subtypes of ICI-related complications, to our knowledge, no study has described the neurological complications occurring in European countries. Therefore, the aim of the present study is to evaluate ICI-related neurological complications reported from the European clinical practice context through the analysis of the pharmacovigilance EudraVigilance database.

## 2 Materials and Methods

### 2.1 Data Source

We retrospectively evaluated all cases of neurologic complications occurring in patients treated with at least one ICI currently authorized by the EMA and collected in EudraVigilance (EV). The EV is the European pharmacovigilance database, managed by the European Medicines Agency (EMA). It is used for the management, collection, and analyses of individual case safety reports (ICSRs) related to both medicines or vaccines (23). The EV contains all ICSRs reported by a healthcare professional or a non-healthcare professional (e.g., citizen or other professional figures) (24). The ICSRs are aimed to inform the competent authorities or a marketing authorization holder regarding the suspected adverse drug reactions (ADRs) or the adverse events following immunization (AEFI) which, in the reporters’ opinion, may be related to assumption of the medicines (drug or vaccine, respectively). The analysis of national and (even more) international databases allows the constant drug safety monitoring processes through the extrapolation of important safety information and signals (25–27). According to a transparency policy, the data are publicly available through the EMA website (www.adrreports.eu). There are different levels of completeness of the available data, some of which require specific authorizations from the Agency. So, we retrieved from the EV database all ICSRs reporting ICI-induced neurologic ADRs (SOC MedDRA “Neurologic disorder”) collected from January 2010 until February 7, 2020.

### 2.2 Descriptive Analysis

The neurologic adverse events were classified according to the Medical Dictionary for Regulatory Activities (MedDRA). MedDRA consists of a five-level hierarchical structure organized like a matryoshka system, and the observations from clinical practice are reported in the lowest level term (LLT). Each LLT is linked to an only one “preferred term” (PT). PT represents the next structural level and expresses the clinical observation coded in a specific diagnosis. Several PTs are clustered based upon anatomy, pathology, physiology, and etiology or function criteria in “High-Level Terms” (HLTs). In turn, more HLTs related according to the abovementioned criteria are subordinate to a higher level, defined as “High-Level Group Terms” (HLGTs). Finally, HLGTs referred to the highest level, “System Organ Classes” (SOCs), which provide the broader concept of data (28). We screened all nervous disorders belonging to the “Nervous system” SOC occurring in patients treated with ipilimumab, nivolumab, pembrolizumab, cemiplimab, atezolizumab, durvalumab, avelumab, or combination ICI treatments, and these were collected in the EudraVigilance database.

We analyzed the annual trend for the collection of ICI-induced neurological complications categorized by years and administered ICI treatments. We analyzed our overall results providing information about the neurological complications, in particular regarding the type of events categorized in HLGTs, their outcome, and mortality rates (intended as no. of specific neurological adverse events with fatal outcome/no. of specific neurological adverse events). The outcome of the neurological complications was classified as “recovered/resolved”, “recovering/resolving”, “recovered/resolved with sequelae”, “not recovered/not resolved”, “fatal”, and “unknown”. Moreover, we analyzed the involved ICI treatments and their therapeutic indications, as well as their gender distribution of complications. We examined the overlapping of more neurological complications in the same patients. In particular, we focused on some events of greatest clinical interest, such as encephalopathy, encephalitis, meningitis, seizures, Parkinson’s disease, myasthenia gravis, and Guillain–Barré syndrome. We provided a more detailed description of events belonging to the most described HLGT, classifying the several neurological diagnoses (p-term) in the reference HLTs. To conclude, we researched in the literature the hypothesized mechanisms, which could be behind these important complications.

### 2.3 Statistical Analysis

We used the chi-square test to evaluate if the differences were statistically significant (*p* < 0.05). In order to identify a hypothetical different reporting probability of the neurologic irADR types between the ICI classes, we performed a disproportionality analysis of ICI-induced neurologic irADRs belonging to the first five HLGTs more frequently reported. The reporting odds ratio (ROR) with 95% CI was computed comparing the different ICI classes to each other based on their pharmacological target (anti-PD-1 vs. anti-PD-L1 vs. anti-CTLA-4). The signal was considered as statistically significant when the lower limit of 95% CI of a ROR exceeded 1.0 (29).

## 3 Results

### 3.1 Descriptive Analysis

**Figure 1:**
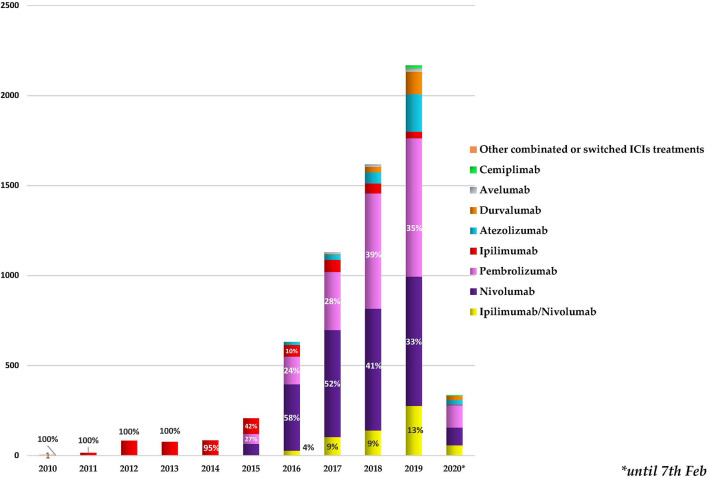
Annual trend for neurologic outcomes occurring in cancer patients treated with one or more immune checkpoint inhibitors (ICIs), categorized by years and administrated ICI treatments. The percentages of the first 3 treatments most involved in the neurological events collected each year are reported. * stands for the date 7th Feb 2021.

As reported in **Figure 1**, we found a growing trend in the number of cases describing ICI-related neurological complications collected in the European database from January 2010. Starting from 2016, more than 500 neurological outcomes/year were collected, exceeding 2,000 events/year in 2019. Ipilimumab was the only ICI to be authorized until 2014, and all cases until that date refer to it. Following the marketing authorization of pembrolizumab and nivolumab, in 2015 and 2016, we found an increase in reporting neurological events related to nivolumab administration (from 30% to 58%) and a reduction in those related to ipilimumab (from 42% to 10%). Simultaneously, a growing trend in description of neurological consequences in patients receiving the combination therapy ipilimumab/nivolumab was found. In 2016, this association was held responsible for 4% of the reported neurological complications related to ICI treatments. This percentage was increased to 13% in 2019. Starting from 2015, other few cases of neurological outcomes occurring in patients treated with other ICI associations not authorized (*N* = 48) were also collected. For example, we found a recent case (collected in 2019) describing an autoimmune encephalitis occurring in an adult male patient affected by malignant melanoma. In this reporting case, four different ICIs (pembrolizumab, nivolumab, ipilimumab, atezolizumab) plus another anticancer agent, the MEK inhibitor cobimetinib, were indicated as suspected drugs. Since in these 48 cases the timing of the different treatments was not specified, it was not possible to define whether the treatments were associated or interchanged with each other. Therefore, we have categorized these cases all together as “other combinations or switched ICI treatments”.

In our study period, overall, 4,875 cases describing 6,429 neurological events as suspected complications of ICI treatments were collected in the EudraVigilance database. Overall, these events were mainly related to the anti-PD-1 agents nivolumab (*N* = 2,520; 39%) and pembrolizumab (*N* = 2,069; 32%), followed by the combination therapy ipilimumab/nivolumab (*N* = 609; 10%), ipilimumab (*N* = 602; 9%), or atezolizumab (*N* = 343; 5%) (**Table 1**).

**Table 1:** Cases describing neurological events occurring in patients receiving at least one ICI collected in EudraVigilance and categorized by pharmacological treatments.

ICI targets	ICI therapies	Cases	Neurological complications
		*N* = 4,875	*N* = 6,429
PD-1	Nivolumab	1,849 (38%)	2,520 (39%)
	Pembrolizumab	1,515 (31%)	2,069 (32%)
CTLA-4	Ipilimumab	582 (12%)	602 (9%)
CTLA-4/PD-1	Ipilimumab/nivolumab	457 (9%)	609 (10%)
PD-L1	Atezolizumab	272 (6%)	343 (5%)
	Durvalumab	122 (3%)	180 (3%)
	Avelumab	33 (0.7%)	40 (0.7%)
PD-1	Cemiplimab	12 (0.3%)	18 (0.4%)
CTLA-4/PD-1 PD-1/PD-L1	Other combinations or switched ICI therapies	34 (0.7%)	48 (0.9%)

CTLA-4, cytotoxic T-lymphocyte antigen-4; ICIs, immune checkpoint inhibitors; PD-1, programmed death-1; PD-L1, programmed death-ligand 1.

Regarding the distribution of cases by therapeutic indications (**Table 2**), ICIs were mainly used for the treatment of lung cancer (1,719/4,875; 35.26%), melanoma (1,293/4,875; 26.52%), or genitourinary tract neoplasm (577/4,875; 11.83%). In 505 cases, the therapeutic indication was not specified (10%). The remaining cases included several types of solid tumors.

**Table 2:** Distribution of therapeutic indication ICI therapies involved in the neurological complications collected in the European database EudraVigilance until February 7, 2020.

Therapeutic indications	ICSRs	Atezolizumab	Avelumab	Cemiplimab	Durvalumab	Ipilimumab	Nivolumab	Pembrolizumab	Ipilimumab/nivolumab	Other ICI combinations
	*N* = 4,875	*N* = 272	*N* = 33	*N* = 12	*N* = 122	*N* = 582	*N* = 1,849	*N* = 1,514	*N* = 457	*N* = 34
	(100%)	(5.58%)	(0.68%)	(0.25%)	(2.5%)	(11.94%)	(37.93%)	(31.06%)	(9.37%)	(0.7%)
Lung cancer	1,719	163	1	–	83	2	781	670	17	2
	(35.26%)	(59.92%)	(3.03%)		(68.03%)	(0.34%)	(42.24%)	(44.25%)	(3.72%)	(5.88%)
Melanoma	1,293	4	–	–	–	396	259	328	280	26
	(26.52%)	(1.47%)				(68.16%)	(14.01%)	(21.66%)	(61.27%)	(76.47%)
Genitourinary tract neoplasm	577	47	5	–	1	8	290	116	109	1
	(11.83%)	(17.28%)	(15.15%)		(0.82%)	(1.37%)	(15.7%)	(7.66%)	(23.85%)	(2.94%)
Unknown indication	505	24	5	3	14	112	146	170	29	2
	(10.35%)	(8.82%)	(15.15%)	(25%)	(11.47%)	(19.27%)	(7.9%)	(11.23%)	(6.34%)	(5.88%)
Gastrointestinal tract cancer	150	4	–	–	1	–	117	27	1	–
	(3.07%)	(1.47%)			(0.82%)		(6.33%)	(1.8%)	(0.22%)	
Head and neck cancer	105	1	2	–	1	–	75	25	1	–
	(2.15%)	(0.37%)	(6.06%)		(0.82%)		(4.06%)	(1.65%)	(0.22%)	
Skin Cancer	67	–	15	7	–	2	24	16	2	1
	(1.37%)		(45.45%)	(58.33%)		(0.34%)	(1.3%)	(1.06%)	(0.44%)	(2.94%)
Lymphoma	67	–	–	–	1	52	13	1	0	–
	(1.37%)				(0.82%)	(8.95%)	(0.7%)	(0.07%)		
Unspecified cancer	56	3	–	1	–	1	30	18	3	–
	(1.15%)	(1.1%)		(8.33%)		(0.17%)	(1.62%)	(1.2%)	(0.66%)	
Breast cancer	49	20	1	–	2	–	5	21	–	–
	(1%)	(7.35%)	(3.03%)		(1.64%)		(0.27%)	(1.4%)		
Female reproductive neoplasm	41	3	2	–	–	–	8	27	1	–
	(0.84%)	(1.1%)	(6.06%)				(0.43%)	(1.8%)	(0.22%)	
Nervous system cancer	39	–	–	1	–	1	25	7	5	–
	(0.8%)			(8.33%)		(0.17%)	(1.35%)	(0.46%)	(1.1%)	
Hepatic and bile duct cancer	36	–	–	–	1	3	24	7	1	–
	(0.74%)				(0.82%)	(0.52%)	(1.31%)	(0.46%)	(0.22%)	
Respiratory tract cancer (excl. lung cancer)	36	1	–	–	1	–	22	11	1	–
	(0.74%)	(0.37%)			(0.82%)		(1.21%)	(0.73%)	(0.22%)	
Mesothelioma	33	–	–	–	–	–	10	20	3	–
	(0.68%)						(0.54%)	(1.32%)	(0.66%)	
Pancreatic cancer	31	–	–	–	16	–	2	13	–	–
	(0.63%)				(13.11%)		(0.11%)	(0.86%)		
Neuroendocrine cancer	29	1	1	–	–	–	5	18	2	2
	(0.6%)	(0.37%)	(3.03%)				(0.27%)	(1.2%)	(0.44%)	(5.88%)
Metastases	16	–	–	–	–	3	4	9	–	–
	(0.33%)					(0.52%)	(0.22%)	(0.6%)		
Leukemia	9	1	1	–	–	2	1	3	1	–
	(0.18%)	(0.37%)	(3.03%)			(0.34%)	(0.05%)	(0.21%)	(0.22%)	
Other indication^a^	5	–	–	–	–	–	2	3	–	–
	(0.1%)						(0.1%)	(0.21%)		
Sarcoma	5	–	–	–	1	–	1	2	1	–
	(0.1%)				(0.82%)		(0.05%)	(0.13%)	(0.22%)	
More of an indication^b^	3	–	–	–	–	–	3	–	–	–
	(0.06%)						(0.16%)			
Bone cancer	3	–	–	–	–	–	1	2	–	–
	(0.06%)						(0.05%)	(0.13%)		
Mediastinum neoplasm	1	–	–	–	–	–	1	–	–	–
	(0.02%)						(0.05%)			

a “Other indication” includes multiple sclerosis, osteoporosis, progressive multifocal leukoencephalopathy, white blood cell count decreased, and mismatch repair cancer syndrome.

b “More of an indication” includes “Brain neoplasm, malignant melanoma, non-Hodgkin’s lymphoma”, “endometrial cancer, metastases to lung, ovarian cancer”, and “Malignant melanoma, papillary thyroid cancer”.

**Figure 2:**
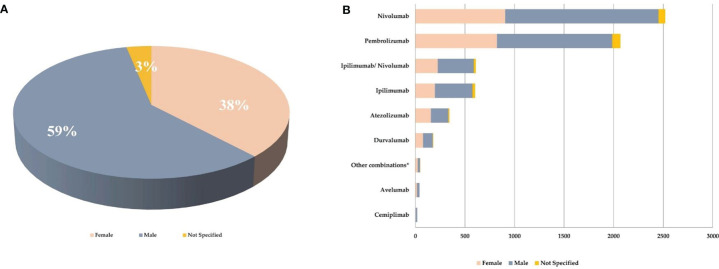
**(A)** Gender distribution of neurological consequences occurring in European patients treated with at least one ICI and collected from the EudraVigilance database until February 7, 2020. **(B)** Gender distribution of neurological outcomes collected from EudraVigilance categorized by single ICI treatment. *pembrolizumab/nivolumab/ipilimumab, pembrolizumab/atezolizumab, ipilimumab/pembrolizumab.

**Figure 3:**
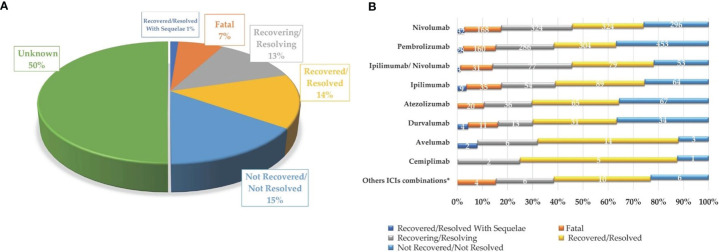
Neurologic complications occurring in European cancer patients treated with one or more ICIs, collected in EudraVigilance until February 7, 2020, categorized by final outcome **(A)** and involved ICI treatment, excluding unknown outcomes **(B)**.

As reported in **Figure 2A**, majority of the reported ICI-related neurological consequences occurred in male patients (*N* = 3,787; 59%) than in female patients (*N* = 2,434; 38%). Analyzing data for each ICI, this gender distribution persisted (**Figure 2B**) [*χ* ^2^ (16, *N* = 6,429) = 42.9; *p* = 0.0003]. In particular, considering ICIs associated with a substantial number of neurological events (≥300), the gender differences were slightly accentuated for ipilimumab (*N* = 602; 63%M vs. 33%F; Δ% = 30; *p* = 0.013), nivolumab (*N* = 2,520; 61%M vs. 36%F; Δ% = 25; *p* = 0.005), and the nivolumab/ipilimumab combination therapy (*N* = 609; 60%M vs. 37%F; Δ% = 23; *p* = 0.668), while these were slightly less evident for pembrolizumab (*N* = 2,069; 56%M vs. 40%F; Δ% = 16; *p* = 0.011) and atezolizumab (*N* = 343; 51%M vs. 46%F; Δ% = 5; *p* = 0.002). No substantial differences emerged in the type of neurological complication in male or female patients and neither in terms of described neurological event nor in terms of reference HLGTs (**Table 3**). The neurological events reported in male patients were more heterogeneous and varied, including 326 different diagnoses compared with 281 diagnoses described for female patients. Finally, although with slight differences in terms of percentages, the neurological consequences mainly reported both in male and female patients were myasthenia gravis (6.4%M vs. 5.2%F), headache (6.1%M vs. 8.8%F), and peripheral neuropathy (5.5M vs. 4.3%F). Instead, slight gender differences emerged regarding the occurrence of tremor and cerebral hemorrhage which were more frequently described in men (2.6%M vs. 1.8%F; 2.1%M vs. 1.5%F, respectively), while somnolence was reported more frequently in women (1.9%M vs. 2.3%F). Although the outcome was unknown for half of the collected neurological events related to ICI treatments (**Figure 3A**), 14% of the ICI-related neurological consequences had a complete resolution and 13% were in resolution, while 23% had unfavorable fallouts, including fatal (7%), not resolved events (15%), or resolved with sequalae (1%). In particular, among 429 events with fatal outcomes, myasthenia gravis (*N* = 49), cerebral hemorrhage (*N* = 34), and cerebrovascular accident (*N* = 25) were the events most frequently described. At the same time, myasthenia gravis (*N* = 10), cerebral hemorrhage (*N* = 10), and cerebral infarction (*N* = 7) were neurological events that more frequently progressed in resolution with sequelae, while neuropathy peripheral (*N* = 74), headaches (*N* = 56), and myasthenia gravis (*N* = 48) were the events mainly reported with no resolution. Regarding the distribution of outcomes categorized for ICI treatments (**Figure 3B**), nivolumab and pembrolizumab were the ICI treatments mostly involved in the reported fatal cases (168/429 and 160/429, respectively). Moreover, pembrolizumab was mostly involved in not resolved neurological events when compared with nivolumab (453 vs. 296 events).

**Table 3:** Top 10 neurological complications and HLGTs more frequently described and collected in the EudraVigilance, categorized by gender.

Neurological complications described in female patients (TOT = 2,434)	*N*	(%)	Neurological complication described in male patients (TOT = 3,769)	*N*	(%)
1. Headache	214	(8.8)	1. Myasthenia gravis	242	(6.4)
2. Myasthenia gravis	126	(5.2)	2. Headache	230	(6.1)
3. Neuropathy peripheral	104	(4.3)	3. Neuropathy peripheral	207	(5.5)
4. Dizziness	98	(4.0)	4. Dizziness	147	(3.9)
5. Seizure	76	(3.1)	5. Seizure	119	(3.1)
6. Cerebrovascular accident	62	(2.5)	6. Tremor	98	(2.6)
7. Hypoesthesia	57	(2.3)	7. Guillain–Barré syndrome	94	(2.5)
8. Somnolence	57	(2.3)	8. Cerebral hemorrhage	81	(2.1)
9. Guillain–Barré syndrome	48	(2.0)	9. Hypoesthesia	81	(2.1)
10. Cerebral infarction	47	(1.9)	10. Cerebral infarction	78	(2.1)
Reference HLGTs of neurological complications described in female patients	*N*	(%)	Reference HLGTs of neurological complications described in male patients	*N*	(%)
1. Neurological disorders NEC	808	(33.1)	1. Neurological disorders NEC	1,210	(32.1)
2. Peripheral neuropathies	249	(10.2)	2. Peripheral neuropathies	482	(12.7)
3. Central nervous system vascular disorders	238	(9.8)	3. Central nervous system vascular disorders	352	(9.3)
4. Headaches	220	(9.1)	4. Neuromuscular disorders	336	(9.0)
5. Neuromuscular disorders	179	(7.3)	5. Headaches	243	(6.4)
6. Movement disorders (incl. parkinsonism)	136	(5.6)	6. Movement disorders (incl. parkinsonism)	231	(6.1)
7. Seizures (incl. subtypes)	111	(4.6)	7. Central nervous system infections and inflammations	184	(4.8)
8. Central nervous system infections and inflammations	109	(4.5)	8. Seizures (incl. subtypes)	173	(4.6)
9. Mental impairment disorders	89	(3.6)	9. Cranial nerve disorders (excl. neoplasms)	145	(3.8)
Cranial nerve disorders (excl. neoplasms)10.	84	(3.4)	Mental impairment disorders10.	123	(3.2)

NEC, not elsewhere classified; HLGTs, High-Level Group Terms.

**Figure 4:**
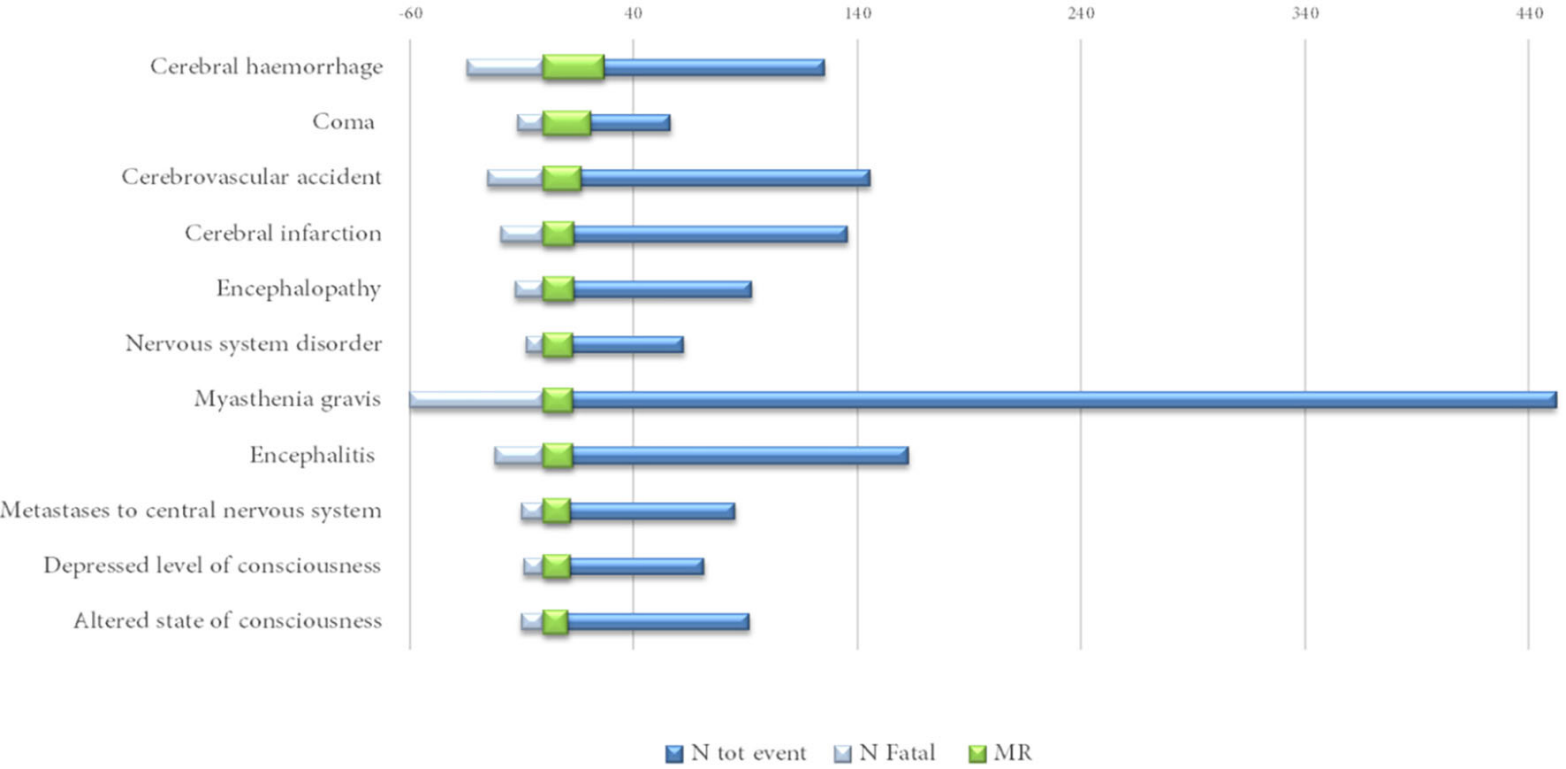
Neurological complications reported in at least 40 cases with a mortality rate (MR) above 10%.

Excluding the infrequently described neurological events (<40 cases), cerebral hemorrhage (MR = 27%), encephalopathy (MR = 14%) and encephalitis and myasthenia gravis (MR = 13%, both) were among those characterized by a mortality rate above 10% (**Figure 4**).

Analyzing data by HLGTs (**Table 4**), the majority of neurological consequences were categorized as “Neurological disorders NEC” (*N* = 2,076; 32%), “Peripheral neuropathies” (*N* = 742; 12%), and “Central nervous system vascular disorders” (*N* = 625; 10%) MedDRA HLGTs. We reported the top 10 diagnoses belonging to the 3 top HLGTs categorized for the involved ICI treatments in **Table 5**. Nivolumab and pembrolizumab were the treatments mainly involved in all HLGTs. The main characteristics of the events belonging to the most represented HLGT, “Neurological disorders NEC”, are described separately in the next subsection.

**Table 4:** Neurologic outcomes occurring in European cancer patients treated with one or more ICIs, collected in EudraVigilance until February 7, 2020, categorized by HLGTs and administered ICI treatments.

HLGT	All ICIs (*N* = 6,429)	Nivolumab (*N* = 2,520)	Pembrolizumab (*N* = 2,069)	Ipilimumab/nivolumab (*N* = 609)	Ipilimumab (*N* = 602)	Atezolizumab (*N* = 343)	Durvalumab (*N* = 180)	Other combinations or switched ICI treatments (*N* = 48)	Avelumab (*N* = 40)	Cemiplimab (*N* = 18)
	*n* (%)	*n* (%)	*n* (%)	*n* (%)	*n* (%)	*n* (%)	*n* (%)	*n* (%)	*n* (%)	*n* (%)
Neurological disorders NEC	2,066 (32.1)	850 (33.8)	683 (33.2)	163 (27.4)	144 (23.8)	114 (33.5)	76 (42.2)	9 (16.7)	21 (55)	6 (33.3)
Peripheral neuropathies	763 (11.9)	272 (10.8)	200 (9.7)	94 (15.4)	115 (19.1)	52 (15.2)	17 (9.4)	10 (20.8)	2 (5)	1 (5.6)
Central nervous system vascular disorders	624 (9.7)	244 (9.7)	224 (10.8)	39 (6.4)	58 (9.6)	27 (7.9)	25 (13.9)	4 (8.3)	2 (5)	1 (5.6)
Neuromuscular disorders	530 (8.2)	218 (8.7)	206 (10)	48 (7.9)	27 (4.5)	16 (4.7)	9 (5)	5 (10.4)	1 (2.5)	
Headaches	474 (7.4)	163 (6.5)	112 (5.4)	69 (11.3)	93 (15.4)	23 (6.7)	8 (4.4)	3 (6.3)	2 (5)	1 (5.6)
Movement disorders (incl. parkinsonism)	382 (5.9)	170 (6.7)	116 (5.6)	21 (3.4)	29 (4.8)	19 (5.5)	18 (10)	–	8 (20)	1 (5.6)
Central nervous system infections and inflammations	299 (4.7)	112 (4.4)	92 (4.4)	41 (6.7)	11 (1.8)	33 (9.6)	5 (2.8)	4 (8.3)	–	1 (5.6)
Seizures (incl. subtypes)	294 (4.6)	115 (4.6)	99 (4.8)	22 (3.6)	25 (4.2)	19 (5.5)	5 (2.8)	5 (10.4)	–	4 (22.2)
Cranial nerve disorders (excl. neoplasms)	233 (3.6)	80 (3.2)	82 (4)	21 (3.4)	31 (5.1)	13 (3.8)	2 (1.1)	4 (8.3)	–	–
Mental impairment disorders	215 (3.4)	79 (3.1)	86 (4.2)	16 (2.6)	15 (2.5)	6 (1.7)	5 (2.8)	4 (8.3)	1 (2.5)	3 (16.7)
Encephalopathies	191 (3)	79 (3.1)	58 (2.8)	19 (3.1)	20 (3.3)	10 (2.9)	4 (2.2)	1 (2.1)	–	–
Increased intracranial pressure and hydrocephalus	123 (1.9)	45 (1.8)	40 (1.9)	26 (4.3)	9 (1.5)	2 (0.6)	1 (0.6)	–	–	–
Spinal cord and nerve root disorders	71 (1.2)	21 (0.8)	25 (1.2)	8 (1.3)	8 (1.3)	4 (1.2)	3 (1.7)	–	2 (5)	–
Demyelinating disorders	54 (0.8)	20 (0.8)	15 (0.7)	5 (0.8)	12 (2)	2 (0.6)	–	–	–	–
Sleep disturbances (incl. subtypes)	30 (0.4)	13 (0.5)	11 (0.5)	5 (0.8)	–	–	1 (0.6)	–	–	–
Neurological disorders of the eye	28 (0.4)	17 (0.7)	4 (0.2)	4 (0.7)	3 (0.5)	–	–	–	–	–
Nervous system neoplasms malignant and unspecified NEC	21 (0.3)	10 (0.4)	7 (0.3)	2 (0.3)	1 (0.2)	–	1 (0.6)	–	–	–
Structural brain disorders	20 (0.3)	9 (0.4)	5 (0.2)	2 (0.3)	2 (0.3)	2 (0.6)	–	–	–	–

NEC, not elsewhere classified; HLGTs, High-Level Group Terms.

**Table 5:** Top 10 ICI-related neurological complications belonging to the three most described High-Level Group Terms (HLGTs) in EudraVigilance until February 7, 2020, categorized by ICI treatments involved.

	Level	All ICIs	Anti-CTLA-4	Anti-PD-1			Anti-PD-L1			Combination therapies	
		*N* = 6,429	Ipilimumab	Nivolumab	Pembrolizumab	Cemiplimab	Durvalumab	Avelumab	Atezolizumab	Ipi/nivo	Others^*^
		(100%)	*N* (%)	*N* (%)	*N* (%)	*N* (%)	*N* (%)	*N* (%)	*N* (%)	*N* (%)	*N* (%)
*HLGT*	“Neurological disorders NEC”	2,066 (32.1)	144 (7)	850 (41.1)	683 (33.1)	6 (0.3)	76 (3.7)	21 (1)	114 (5.5)	163 (7.9)	9 (0.4)
	1. Dizziness	250 (12.1)	23 (9.2)	102 (40.8)	80 (32)	–	8 (3.2)	5 (2)	13 (5.2)	17 (6.8)	2 (0.8)
	2. Hypoesthesia	140 (6.8)	13 (9.3)	48 (34.3)	49 (35)	–	4 (2.9)	1 (0.7)	10 (7.1)	15 (10.7)	–
	3. Somnolence	130 (6.2)	5 (3.8)	49 (37.7)	46 (35.4)	–	5 (3.8)	3 (2.3)	9 (7)	13 (10)	–
	4. Loss of consciousness	108 (5.2)	6 (5.6)	39 (36.1)	37 (34.2)	1 (1)	1 (1)	1 (0.9)	10 (9.2)	13 (12)	–
	5. Paraesthesia	105 (5.1)	8 (7.6)	49 (46.7)	28 (26.7)	–	4 (3.8)	–	7 (6.7)	9 (8.5)	–
	6. Altered state of consciousness	92 (4.5)	3 (3.3)	36 (39.1)	34 (37)	–	1 (1.1)	2 (2.2)	7 (7.6)	6 (6.5)	3 (3.2)
	7. Metastases to CNS	86 (4.2)	5 (5.8)	47 (54.6)	10 (11.6)	–	13 (15.1)	–	1 (1.2)	9 (10.5)	1 (1.2)
	8. Syncope	81 (4)	9 (11.1)	29 (35.8)	25 (30.9)	1 (1.2)	3 (3.7)	2 (2.5)	5 (6.2)	7 (8.6)	–
	9. Dysgeusia	72 (3.5)	2 (2.8)	32 (44.4)	31 (43)	–	1 (1.4)	–	4 (5.6)	2 (2.8)	–
	10. Depressed level of consciousness	72 (3.5)	1 (1.4)	31 (43)	25 (34.7)	–	4 (5.6)	1 (1.4)	7 (9.7)	3 (4.2)	–
*HLGT*	“Peripheral neuropathies”	773 (12)	115 (14.9)	276 (35.7)	204 (26.4)	1 (0.1)	17 (2.2)	3 (0.4)	52 (6.7)	95 (12.3)	10 (1.3)
	1. Neuropathy peripheral	335 (43.3)	49 (14.6)	120 (35.8)	90 (26.8)		8 (2.4)	33 (9.8)	2 (0.6)	30 (9)	3 (1)
	2. Guillain–Barré syndrome	154 (20)	29 (18.8)	46 (30)	41 (26.6)		2 (1.3)	7 (4.5)		25 (16.2)	4 (2.6)
	3. Polyneuropathy	69 (9)	10 (14.5)	24 (34.8)	16 (23.2)	1 (1.4)	2 (2.9)	4 (5.8)		11 (16)	1 (1.4)
	4. Peripheral sensory neuropathy	42 (5.4)	5 (11.9)	17 (40.5)	15 (35.7)		3 (7.1)	1 (2.4)		1 (2.4)	
	5. Demyelinating polyneuropathy	29 (3.8)	6 (20.7)	11 (38)	5 (17.2)	–	–	2 (6.9)	–	5 (17.2)	
	6. Neuritis	19 (2.5)	1 (5.3)	9 (47.4)	4 (21)	–	–	1 (5.3)	–	4 (21)	
	7. Autoimmune neuropathy	18 (2.3)	2 (11.1)	6 (33.3)	4 (22.2)	–	1 (5.6)	–	–	5 (27.8)	
	8. Carpal tunnel syndrome	13 (1.7)	1 (7.7)	7 (53.8)	5 (38.5)	–	–	–	–		
	9. Chronic inflammatory demyelinating polyradiculoneuropathy	12 (1.5)	2 (16.7)	4 (33.3)	3 (25)	–	–	–	–	2 (16.7)	1 (8.3)
	10. Peripheral motor neuropathy	11 (1.4)	1 (9.1)	5 (45.4)	4 (36.4)	–	–	–	–	1 (9.1)	
*HLGT*	“Central nervous system vascular disorders”	624 (9.7)	58 (9.3)	244 (39.1)	224 (36)	1 (0.2)	25 (4)	2 (0.3)	27 (4.3)	39 (6.2)	4 (0.6)
	1. Cerebrovascular accident	146 (23.4)	16 (11)	48 (32.9)	56 (38.3)	–	8 (5.5)	–	10 (6.8)	7 (4.8)	1 (0.7)
	2. Cerebral infarction	128 (20.5)	–	48 (37.5)	69 (54)	–	4 (3.1)	–	2 (1.5)	5 (3.9)	–
	3. Cerebral hemorrhage	126 (29.2)	21 (16.7)	49 (38.9)	29 (23)	–	3 (2.4)	–	5 (4)	19 (15)	–
	4. Ischemic stroke	36 (5.8)	2 (5.5)	19 (52.8)	12 (33.3)	–	1 (2.8)	–	1 (2.8)	1 (2.8)	–
	5. Hemorrhage intracranial	25 (4)	6 (24)	10 (40)	6 (24)	–	–	1 (4)	1 (4)	1 (4)	–
	6. Transient ischemic attack	23 (3.7)	1 (4.3)	12 (52.3)	4 (17.5)	1 (4.3)	1 (4.3)	–	1 (4.3)	1 (4.3)	2 (8.7)
	7. Cerebral ischemia	17 (2.7)	2 (11.8)	6 (35.3)	5 (29.4)	–	3 (17.6)	–	–	1 (5.9)	–
	8. Lacunar infarction	10 (1.6)	1 (10)	4 (40)	5 (50)	–	–	–	–	–	–
	9. Subarachnoid hemorrhage	9 (1.4)	1 (11.1)	7 (77.8)	1 (11.1)	–	–	–	–	–	–
	10. Vasculitis cerebral	9 (1.4)	–	5 (55.6)	3 (33.3)	–	–	1 (11.1)	–	–	–

NEC, not elsewhere classified; HLGTs, High-Level Group Terms.

*stand for pembrolizumab/nivolumab/ipilimumab, pembrolizumab/atezolizumab, ipilimumab/pembrolizumab.

**Figure 5:**
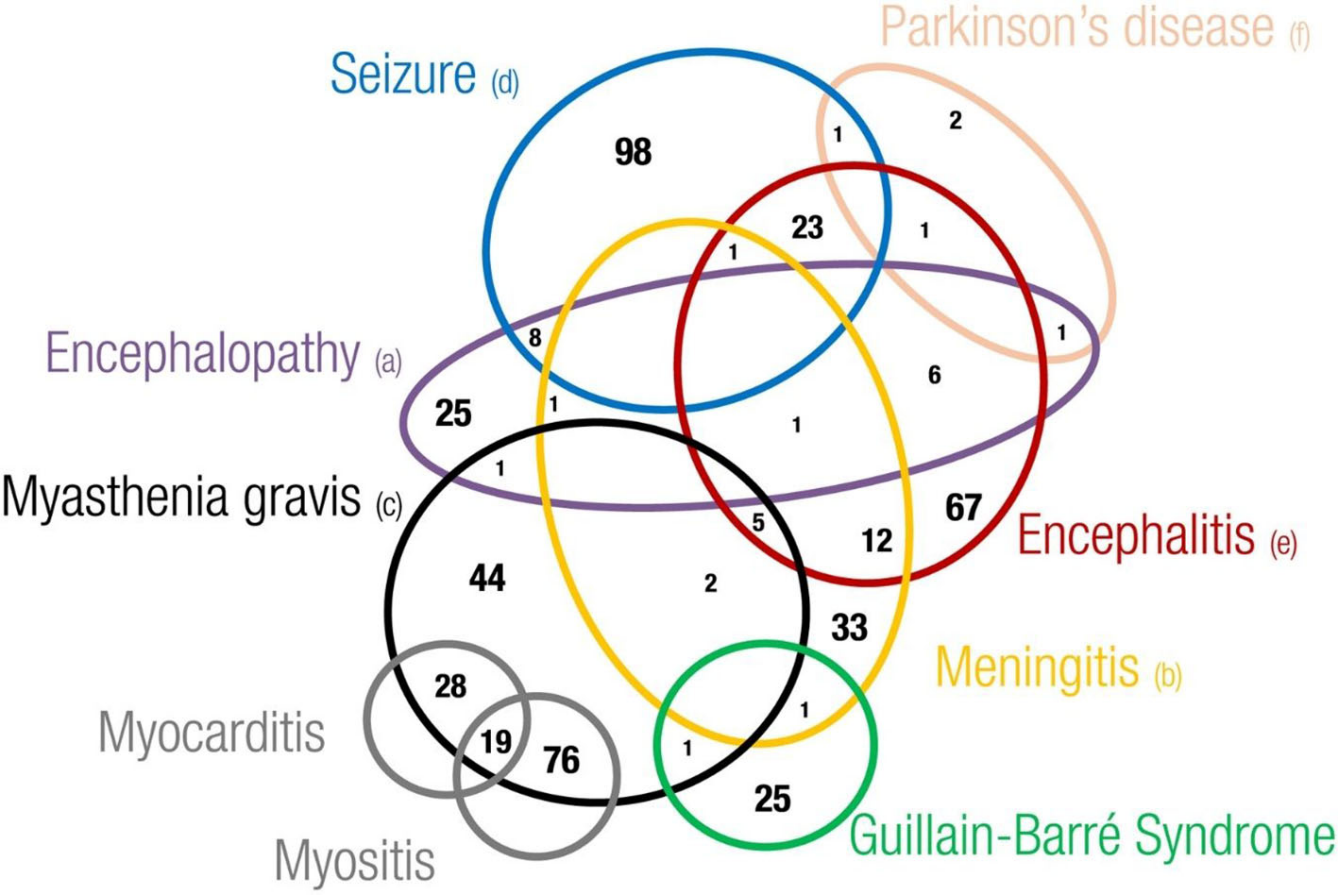
Eulero Venn diagram showing overlapping between neurotoxicities of main interest. (a) Encephalopathy includes leukoencephalopathy hypoxic-ischaemic encephalopathy, encephalopathy, hepatic ence-phalopathy, posterior reversible encephalopathy syndrome and autoimmune encephalopathy (b) Meningitis includes meningism, meningitis, meningitis aseptic, meningitis tuberculous, meningitis noninfective, meningeal disorder, meningitis listeria, meningitis viral and pachymeningitis (c) Myasthenia gravis includes myasthenia gravis and mya-sthenic syndrome (d) Seizure includes epilepsy, clonus, myoclonus, partial seizures, generalised tonic-clonic seizure, seizure, seizure like phenomena, status epilecticus and tonic convulsion (e) Encephalitis includes encephalitis, ence-phalitis toxic, encephalitis autoimmune, limbic encephalopathy and noninfective encephalitis (f) Parkinson's disease includes Parkinson's disease and parkinsonism.

Regarding the overlapping of neurological irAEs, we found that 1,094 out of 4,875 cases described more than one neurological complication (22%). Overall, we found a total of 2,647 out of 6,429 overlapping neurological events (41.2%). Headache, dizziness (including the postural type), encephalitis, metastases to the central nervous system (CNS), and convulsive states were the complications more frequently overlapping with other neurological events. Although to a lesser extent, other important neurological complications involved in some overlapping cases were myasthenia gravis, meningitis, memory impairment, speech disorders, encephalopathy, and Guillain–Barré syndrome (data not shown). Examining the overlapping of neurological adverse events of major interest, we found that seizures (*N* = 131), encephalitis (*N* = 117), and meningitis (*N* = 56) were the neurological complications more frequently overlapping with other neurological events (**Figure 5**). These were rarely overlapping with each other, except for encephalitis with seizure (*N* = 23) or with meningitis (*N* = 12). In addition, we noticed a recurrent overlapping of myasthenia gravis and myositis (*N* = 76) or myocarditis (*N* = 28), or both (*N* = 19).

#### 3.1.1 Neurological Disorders NEC

“Neurological disorders NEC” represents a large and heterogeneous group of nervous complications not elsewhere classified (NEC). In our analysis, this was the most represented HLGT, accounting for 32% of the overall ICI-related neurological outcomes described in the European database in our study period.

Overall, we found 2,066 ICI-related neurological complications categorized in this HLGT, counting 104 different diagnoses. These diagnoses were categorized into 12 different HLTs (**Table S1**). “Disturbances in consciousness” (*N* = 566; 26.7%), including somnolence (*N* = 130), loss and altered state of consciousness (*N* = 198 and *N* = 92, respectively), and syncope (*N* = 81), was the most representative HLT. Although to a lesser extent, disturbances of coordination and balance, coma states, and speech and language dysfunctions were also described. Regardless of the HLT categorization, dizziness (*N* = 250), hypoesthesia (*N* = 140), and somnolence (*N* = 130) were the neurological diagnoses belonging to this HLGT, which were more frequently described in EudraVigilance, representing overall the 25% of the complications included in this HLGT (520/2,066). The onset of dizziness and somnolence was mainly related to nivolumab administration (*N* = 102, 40.8%, and *N* = 49, 37.7%, respectively). Instead, hypoesthesia was mainly reported in patients treated with pembrolizumab (*N* = 49; 35%). There were also six described cases of paraneoplastic neurological syndromes related to the anti-PD-1 agents pembrolizumab and nivolumab. Moreover, the onset of metastases to the nervous system was reported in 97 cases, of which 11 cases were reported to have metastases of the meninges. The reported metastases to the nervous system were mainly related to nivolumab (*N* = 57; 58.7%), followed by durvalumab (*N* = 13, 13.4%) and pembrolizumab (*N* = 10, 10.3%). Although more cases were associated with nivolumab, the reporting percentage of this complication was higher for durvalumab (3.1% vs. 10.7%). Metastases to the nervous system were always overlapping with other events. Specifically, cerebral hemorrhage (*N* = 16), headache (*N* = 14), seizure (*N* = 9), and brain edema and dizziness (*N* = 8, both) were the neurologic adverse events more frequently reported with brain metastases. Moreover, in 31 cases, metastases to the nervous system were associated with disease progression or condition aggravated. In six of these cases, ICIs were used for an unapproved indication, including thyroid cancer, gastric cancer, triple negative breast cancer, and neuroendocrine carcinoma of the skin. Moreover, the treatment with ICIs was discontinued in 51.4% of the cases (data not shown).

As reported in **Table 3**, “Neurological disorders NEC” was the most frequent described HLGT both in women (33.1%) and men (32.1%). Although the patient’s sex was not specified for 48 reported disorders (2.3%), the majority of these disorders occurred in male patients than in female patients (58.6%M vs. 39.1%F). Moreover, the described neurological disorders NEC were often serious: 46.2% of these events caused or prolonged hospitalization (*N* = 954), while 36.3% requested the discontinuation of the treatment with ICIs (*N* = 750). Of the described neurological disorders NEC, 14.6% had a complete resolution (*N* = 303), 14.2% were not resolved (*N* = 294), 10.4% were in resolution (*N* = 214), and 0.3% had a resolution with sequelae (*N* = 7). Moreover, 5% of the events had a fatal outcome (*N* = 101). The fatal events were mainly represented by coma states (*N* = 13), metastases to the central nervous system (*N* = 10), and altered (*N* = 10) or depressed level of consciousness (*N* = 9). Fatal consequences occurred mainly in men (*N* = 55; 54.5%) than in women (*N* = 43; 42.6%) and were mainly related to pembrolizumab (55.4%) and nivolumab (28.7%) (data not shown). Other ICI therapies involved in fatal outcomes were ipilimumab/nivolumab association (4.9%), ipilimumab (4%), atezolizumab (3%), and durvalumab (3%). No fatal events were reported following treatments with avelumab or cemiplimab (data not shown).

### 3.2 Disproportionality Analysis

**Figure 6:**
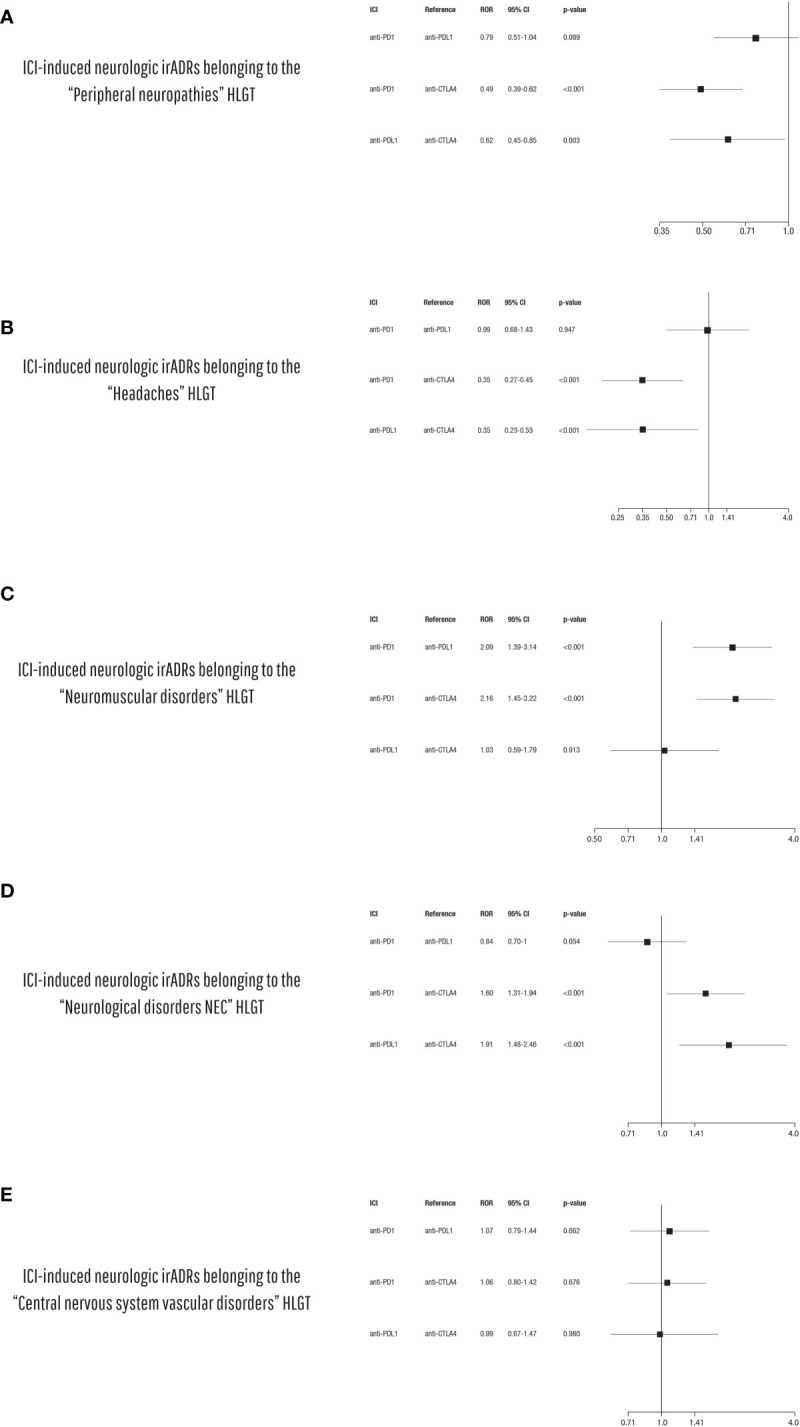
Disproportionality analysis of ICI-induced neurologic irADRs belonging to **(A)** the “Peripheral neuropathies”, **(B)** the “Headaches”, **(C)** the “Neuromuscular disorders”, **(D)** “Neurological disorders NEC”, and **(E)** “Central nervous system vascular disorders” HLGTs comparing the different ICI classes to each other (anti-PD-1 vs. anti-PD-L1 vs. anti-CTLA-4).

Applying the ROR, we found that the anti-CTLA-4 agent ipilimumab was associated with an increased reporting probability of neurologic irADRs belonging to “Peripheral neuropathies” (**Figure 6A**) and “Headaches” HLGTs (**Figure 6B**) when compared with all other ICI classes. Instead, anti-PD-1 agents were associated with an increased reporting probability of neuromuscular disorders rather than other neurologic event types when compared with ICIs targeting PD-L1 and CTLA-4 (**Figure 6C**). Moreover, irADRs belonging to the “Neurologic disorders NEC” HLGT were more probably reported with anti-PD-1 and anti-PD-L1 agents compared with ipilimumab (anti-CTLA-4) (**Figure 6D**). No signal considered statistically significant emerged for irADRs belonging to “Central nervous system vascular disorders” HLGT (**Figure 6E**).

## 4 Discussion

Neurotoxicities related to ICI treatments are various, differing in type and their impact on the patient’s health. They include a wide range of events involving both the central and the peripheral nervous system (2). Although some of the more frequent events (such as headache or peripheral neuropathies) may be considered less clinically relevant, they can equally affect the patient’s quality of life. Furthermore, other possible neurological outcomes consist of serious and potentially devastating events, such as vascular or infectious and inflammatory disorders of the CNS, including cerebral hemorrhages, vasculitis or encephalitis, meningitis, and myelitis (21).

Immunotherapies can induce either new immune-mediated inflammatory events or a flare-up of pre-existing autoimmune disorders involving the nervous system. In our study, we found some cases of myasthenic crisis or relapse of sclerosis multiple. In the literature, several case reports of flare-up or relapses of autoimmune disorders after treatment with ICIs were described. In 2015, Gettings et al. reported a case of severe relapse occurring in a patient affected by metastatic melanoma and concomitant stable relapsing–remitting multiple sclerosis after treatment with ipilimumab (30). Kimura et al. even described a case of a myasthenic crisis and polymyositis arising in an 80-year-old man with multiple lymph node metastasis of malignant melanoma induced by a single dose of nivolumab. According to the authors, the patient was probably already suffering from a subclinical myasthenia gravis, which progressed to myasthenic crisis following a severe autoimmune response induced by nivolumab administration. This case report suggests the importance of conducting appropriate studies in order to exclude the presence of subclinical autoimmune diseases before starting ICI treatment (31).

Although it was not possible from our dataset to extrapolate information regarding time to event of the described complications, several studies have shown that neurological complications generally occur shortly after starting therapy. In fact, based on the results of a recent pharmacovigilance study conducted by Johnson et al. on the World Health Organization database VigiBase, the neurologic outcomes of patients undergoing ICI therapy increased in the first 3 months of therapy (22). In particular, compared with other events, myasthenia gravis was the neurological complication characterized by a more rapid onset and correlated with higher mortality rates.

Similarly, myasthenia was among the events with the highest mortality rate in our dataset. In particular, myasthenia gravis is considered an emerging side effect of immunotherapies (32), and in the literature, there are many case reports that consider myasthenia as a possible complication of anti-PD-1, anti-PD-L1, or anti-CTLA-4 therapy (2, 30, 31, 33–37). This autoimmune neuromuscular disorder, characterized by muscular weakness involving also the respiratory muscles, is due to an immune attack against postsynaptic structures of the neuromuscular junctions (2). According to Psimaras et al., neuromuscular disorders are the most common neurological outcomes related to ICI treatment (11). In line with this, in our analysis, neuromuscular disorders (in particular myasthenia gravis) were among the most frequently reported neurological complications in patients receiving ICI treatments, representing the fourth most frequently described HLGT. Some differences in terms of overall risk, types, and seriousness of ICI-related neurological complication were reported among the different ICI therapeutic regimens.

According to Blackmon et al., neurological phenomenon following immune checkpoint blockade seems to occur more frequently in patients treated with ipilimumab, in mono- or combination therapy (21). Moreover, specific class–event associations were highlighted by Johnson who showed that encephalitis and myasthenia gravis were mainly related to treatments with anti-PD-1/PD-L1 agents, while GBS and meningitis were mainly related to anti-CTLA-4 combination therapies. Likewise, in a Japanese study conducted in 9,800 patients treated with nivolumab and ~400 patients treated with ipilimumab, the complication of myasthenia gravis (MG) has been observed to occur more frequently in patients treated with nivolumab (anti-PD-1) than in those treated with ipilimumab (anti-CTLA-4). Furthermore, comparing their clinical characteristics with a control group of 105 patients with idiopathic type MG, it was found that patients with ICI-induced MG had a higher rate of life-threatening myasthenic seizures needing respiratory support (34). Overall, in our study period, neurological events were mainly related to the anti-PD-1 agents nivolumab and pembrolizumab. In particular, we found a particular association between nivolumab treatments and the CNS ischemic disorders (ischemic stroke and transient ischemic attack) or intracranial hemorrhagic events. We did not find a particular association of specific anti-CTLA-4 events. The type of immune-related neurological complication seems to be also influenced by the type and staging of the neoplastic disease treated with ICIs. In particular, according to Shi et al., patients with melanoma might be at a particularly high risk of such complications (38). In our analysis, the majority of cases reporting neurological outcomes involved patients affected by lung cancer and melanoma. These diseases are those in which ICIs are used more and for a longer time, being the first authorized therapeutic indications shared by the most used ICIs (3, 39). In the same way, it should be considered that melanoma and lung cancer patients commonly present brain metastases such as secondary solid tumor (40–42). So, based on this, the reported brain metastases could also be considered possible therapeutic failure, which today, according to good pharmacovigilance practices (GVP), should be submitted as ICSR when there is at least an associated ADR (43). When CNS metastases occur in patients treated with ICIs for melanoma or lung cancer, the possible role of the underlying disease on the occurrence of these neurological adverse events may be considered. Certainly, this is considered in the case-by-case analysis for the causality assessment, applying specific algorithms like Naranjo’s algorithm. In our opinion, it would be very interesting to perform an analysis on the predisposing factors, but this would require a higher quality of data collection, paying attention to reporting accurate information regarding the concomitant conditions and therapies, which, instead, are often neglected or omitted by the reported (if not solicited). Several studies reported gender differences in the occurrence of ICI complication. In line with our results, this sex-based distribution is dominated by men, amounting to around 60%:40% (men:women) in greater clinical studies (44). To explain this gender difference, several factors should be considered, including the impact of gender on the immune system due to hormonal differences and differences in sex chromosome genes (45, 46). Gender differences in terms of pathogenesis, biology, and epidemiology of cancer diseases, such as those related to lung cancer or melanoma (among the main therapeutic indications of ICIs), are equally important (47, 48). Differences in terms of the tumor environment (warmer in women) and its antigenicity (higher in men) may also have an effect on cancer immunotherapy efficacy (37). Generally, the magnitude of ICI efficacy is sex-dependent, with more favorable results in men, particularly for anti-CTLA-4 agents (45, 49, 50). Some authors have correlated the onset of unwanted autoimmune events with the greater efficacy of immunotherapy. However, concerns have been raised regarding the methodological approach used to produce this evidence; therefore, the data in the literature are still conflicting (51).

According to the analysis of Johnson et al., neurological complications were rarely overlapping (22). It was interesting to note that the overlap of myasthenia with other neurological or non-neurological events induces an increase in the relative mortality rates. In the same way, we noted a frequent overlap of MG with myositis and myocarditis. Recently, describing complex cases of neuromuscular disorders, which included concomitant myasthenia gravis, myositis, myocarditis, and polyneuropathy occurring in patients receiving ICIs, Rota et al. suggest a life-threatening continuum of neuromuscular and cardiac toxicity. Considering these and our several overlapping events, heart and skeletal muscles seem to be concomitant autoimmune targets (52).

Overall, the onset of such unwanted neurological manifestations is related to the pharmacodynamic properties of these drugs. Their pharmacologic targets are involved in maintaining immune tolerance and their actions are not limited to the tumor microenvironment. In fact, neurological complication can be considered as “off-target” effects of ICIs, due to the expression of their ligands in different normal tissues including nervous tissues, like CTLA-4 expressed on pituitary cells or PD-1 on astrocytes and neurons. Actually, evidence concerning the pathogenesis of CNS toxicities following immune checkpoint blockade is growing but yet uncertain. It is well known how the gut microbiota can influence the immune system, influencing its possible response to ICI therapy (53). At same time, the mutual relationship between gut microbiota and neurologic disorders is widely recognized, since gut microbiota can interact with the CNS through the so-called microbiota–gut–brain axis (54, 55). Probably, a genetic predisposition and microbiome alterations can play a role in the development of immune-related adverse events, including neurological complications (56, 57). In a recent real-world study, Wenhui Liu et al. highlighted how the gut microbiome diversity influences the occurrence of ICI-induced irAEs, including peripheral neuritis associated with anti-PD-1 inhibitor use. According to their results, the gut microbiota can be considered as the source for developing predictive biomarkers useful to predict the occurrence of irAEs, especially in terms of event seriousness (53). Moreover, the presence of pre-existing subclinical autoimmunity phenomena might contribute to the occurrence of ICI-related immune complications, including nervous complications (58). The presence of anatomical barriers (such as the blood–brain and blood–nerve barrier), and a different structure of the lymphatic system for the nervous system compared with other organs, could affect the low incidence of ICI-related neurological complications (59). Neurologic phenomena reversibility seems to be influenced by the different pathogenic mechanisms involved (21). To date, several pathogenetic mechanisms have been hypothesized, including cross-presentation of neoantigens, downregulation of regulatory T cells (Treg cells), neuronal damage by effector T cells and autoantibodies, and/or cytokine-mediated inflammation processes. In particular, the first possible mechanism could involve the breaking of immune tolerance related to inhibition of Treg cell actions. In fact, Treg cells, commonly expressed on their surface CTLA-4 and PD-1 receptors, are involved in self-tolerance processes by CD8^+^ T-cell inhibition. Blocking immune checkpoints, the inhibition commonly exerted by Treg cells on effector T cells induces a hyperproliferation and hyperactivation of the latter, breaking the physiological immune tolerance. Another pathogenetic mechanism underlying neurotoxicity seems to be a possible molecular similarity between tumor and self-tissue antigen, called molecular mimicry. This is probably due to shared origins of different cells types from the same embryonic structure. This happens, for example, for melanocytes and Schwann cells which originate from the same cellular precursors of the neural crest. This could explain the occurrence of demyelinating polyneuropathy in melanoma patients treated with ICIs, such as a cross-reactive immune response against the host antigen expressed on normal nervous tissue. Moreover, epitope spreading is another supposed mechanism. It involves an expansion of an immune response against secondary antigens (initially not recognized by the effector T cell) due to their release following tissue damage obtained during the response to immunotherapy (59).

Considering the data source that we used, this study has the intrinsic limitations of the spontaneous reporting systems. Indeed, although they represent the cornerstone of pharmacovigilance activities, the spontaneous reporting systems are characterized by possible underreporting, low quality of data, and lack of data including line treatment and data on drug exposure. Despite these limitations, our data were based on a real-life context, whose importance is recognized as it allows a better characterization of the drug safety profiles, overcoming the well-known intrinsic limits of clinical trials. The analysis of real-life data using large databases, like EudraVigilance, allows to advance hypotheses which need to be confirmed by the results of further studies, including cohort and case–control studies, meta-analysis, and novel bioinformatic methods, which can be extremely valuable in providing new evidence regarding the safety profile of these innovative drugs.

## 5 Conclusions

Since January 2010 until February 2020, ICI-related neurological complications had been increasingly described in the European context of clinical practice. In 2019, more than 2,000 neurological complications arising in patients receiving ICI treatments were collected in the EudraVigilance database. Overall, we found 4,875 cases collected in the European database which described 6,429 neurological events related to ICI treatments. These were mainly related to the anti-PD-1 agents nivolumab and pembrolizumab, followed by the combination therapy ipilimumab/nivolumab and ipilimumab monotherapy. Regardless of the nature of the neurological event and ICI involved, we found a gender distribution with a higher burden for male patients. The neurological events reported in male patients were more heterogeneous and varied than in female patients. Myasthenia gravis is an important neurological consequence often associated with unfavorable outcomes. Growing but yet uncertain evidence regarding pathogenic mechanisms supported the possible relationship between ICI treatments and neurological outcomes, such as peripheral neuropathies (including acute and chronic forms) and cerebrovascular disorders (TIA, ischemic stroke, and cerebral vasculitis). The risk of immune-related complications seems to be the price of durable responses and prolongation of survival allowed by immunotherapies. Considering the recent marketing authorizations of ICIs, further studies are strongly needed to evaluate their neurologic safety profile. There is a strong need to continue to collect, monitor, and analyze data available from the clinical setting (23, 60). It is equally necessary to understand the pathogenic mechanisms underlying these disorders, in order to identify possible biomarkers to prevent them (16). These complications can endanger the lives of patients or impair their daily activities (61). Moreover, such complications can also have a significant economic burden. Champiat et al. included the prevention of dysimmune toxicities in their management model of patients under ICI immunotherapy. Knowledge of the immunotoxicity spectrum, identification of risk factors for dysimmunity, and patient information were among the tools suggested by the authors for the prevention of ICI-related neurological complications (62). However, the identification of specific biomarkers useful for predicting the onset of neurologic irAE still remains a current challenge. Therefore, as specific strategies are not yet available for the prevention of ICI-related NirADRs, oncologists should closely monitor their patients, be prepared to diagnose early the possible ICI-related neurological outcomes in collaboration with neurologists, and treat their patients appropriately in order to avoid serious sequelae and permanent discontinuation of ICI. Moreover, they should inform and educate their patients to immediately report any new symptom or worsening of pre-existing ones during the ICI treatment.

## Data Availability Statement

The datasets presented in this study can be found in online repositories. The names of the repository/repositories and accession number(s) can be found below: www.adrreports.eu.

## Ethics Statement

Ethical review and approval was not required for the study on human participants in accordance with local legislation and institutional requirements. Written informed consent for participation was not required for this study in accordance with national legislation and institutional requirements.

## Author Contributions

Conceptualization: AC and RD. Methodology: AC, RR, and CR. Data curation: BS, FF, RDN, and RR. Writing—original draft preparation: RR, BS, and FF. Writing—review and editing: CS, CR, and LS. Visualization: GdM. Supervision: MDR and FR. Project administration: MDR and RD. Funding acquisition: RD. All authors have read and agreed to the published version of the manuscript.

## Funding

This research was funded under grant no. 2017NR7W5K_002 (PRIN 2017) from MIUR, Italy.

## Conflict of Interest

The authors declare that the research was conducted in the absence of any commercial or financial relationships that could be construed as a potential conflict of interest.

## Publisher’s Note

All claims expressed in this article are solely those of the authors and do not necessarily represent those of their affiliated organizations, or those of the publisher, the editors and the reviewers. Any product that may be evaluated in this article, or claim that may be made by its manufacturer, is not guaranteed or endorsed by the publisher.
