# Supplementary material for: Neurological Manifestations Related to Immune Checkpoint Inhibitors: Reverse Translational Research by Using the European Real-World Safety Data
Source: Front Oncol. 2022 Mar 15;12:824511. doi: 10.3389/fonc.2022.824511 (PMC8964934; doi:10.3389/fonc.2022.824511)
Supplement: Supplementary file 1 [file Table_1.docx]

***Supplemental Digital Content***

**Table S1. Distribution of neurological complications belonging to “Neurological Disorders NEC” HGLT occurred in European patients treated with at least one ICI. The neurological complications were categorized for High Level Terms (HLT) MedDRA and the involved ICI treatments.**

| **High Level Terms** | **All ICIs (N=6249)** | **Ipilimumab (N=602)** | **Nivolumab (N=2520)** | **Pembrolizumab (N=2069)** | **Cemiplimab (N=18)** | **Atezolizumab (N=343)** | **Avelumab (N=40)** | **Durvalumab (N=180)** | **Ipilimumab/ nivolumab  (N=609)** | **Other combination or switched ICIs treatments* (N=48)** |
| --- | --- | --- | --- | --- | --- | --- | --- | --- | --- | --- |
| **Disturbances in consciousness NEC** | **551 (8.8)** | **28 (4.7)** | **212 (8.4)** | **193 (9.3)** | **2 (11.1)** | **42 (12.2)** | **9 (22.5)** | **19 (10.6)** | **43 (7.1)** | **3 (6.3)** |
| Somnolence | 130 | 5 | 49 | 46 | **-** | 9 | 3 | 5 | 13 | **-** |
| Loss of consciousness | 108 | 6 | 39 | 37 | 1 | 10 | 1 | 1 | 13 | - |
| Altered state of consciousness | 92 | 3 | 36 | 34 |  | 7 | 2 | 1 | 6 | 3 |
| Syncope | 81 | 9 | 29 | 25 | 1 | 5 | 2 | 3 | 7 | - |
| Depressed level of consciousness | 72 | 1 | 31 | 25 | - | 7 | 1 | 4 | 3 | - |
| Lethargy | 59 | 4 | 21 | 25 | - | 3 | - | 5 | 1 | - |
| Sedation | 3 | - | 2 | 1 | - | - | - | - | - | - |
| Stupor | 2 | - | 2 | - | - | - | - | - | - | - |
| Apallic syndrome | 1 | - | 1 | - | - | - | - | - | - | - |
| Consciousness fluctuating | 1 | - | 1 | - | - | - | - | - | - | - |
| Sleep deficit | 1 | - | - | - | - | 1 | - | - | - | - |
| Sopor | 1 | - | 1 | - | - | - | - | - | - | - |
| **Neurological signs and symptoms NEC** | **366 (5.9)** | **31 (5.1)** | **135 (5.4)** | **130 (6.3)** | **-** | **19 (5.5)** | **6 (15)** | **15 (8.3)** | **28 (4.6)** | **2 (4.2)** |
| Dizziness | 250 | 23 | 102 | 80 | - | 13 | 5 | 8 | 17 | 2 |
| Neurological symptom | 24 | 3 | 4 | 11 | - | 1 | 1 | - | 4 | - |
| Unresponsive to stimuli | 17 | - | 2 | 12 | - | 2 | - | 1 | - | - |
| Presyncope | 16 | 1 | 4 | 5 | - | 1 |  | 3 | 2 | - |
| Dizziness postural | 12 | - | 3 | 7 | - | - | - | 2 | - | - |
| Myoclonus | 11 | - | 5 | 4 | - | - | - | - | 2 | - |
| Restlessness | 10 | - | 5 | 3 | - | - | - | - | 2 | - |
| Neurological decompensation | 9 | - | 2 | 4 | - | 2 | - | - | 1 | - |
| Head discomfort | 7 | 2 | 2 | 2 | - | - | - | 1 |  | - |
| Cerebrospinal fluid leakage | 3 | - | 2 | 1 | - | - | - | - | - | - |
| Meningism | 2 | 1 | 1 | - | - | - | - | - | - | - |
| Clonus | 1 | - | 1 | - | - | - | - | - | - | - |
| Dizziness exertional | 1 | - | - | 1 | - | - | - | - | - | - |
| Mobility decreased | 1 | - | 1 | - | - | - | - | - | - | - |
| Pleocytosis | 1 | 1 |  | - | - | - | - | - | - | - |
| Drooling | 1 | - | 1 | - | - | - | - | - | - | - |
| **Paraesthesias and dysaesthesias** | **292 (4.7)** | **25 (4.2)** | **119 (4.7)** | **95 (4.6)** | **-** | **18(5.2)** | **1 (2.5)** | **9 (5)** | **25 (4.1)** | **-** |
| Hypoaesthesia | 140 | 13 | 48 | 49 | - | 10 | 1 | 4 | 15 | - |
| Paraesthesia | 105 | 8 | 49 | 28 | - | 7 | - | 4 | 9 | - |
| Burning sensation | 19 | 2 | 6 | 9 | - | - | - | 1 | 1 | - |
| Hyperaesthesia | 8 | - | 5 | 3 | - | - | - | - | - | - |
| Dysaesthesia | 5 | - | 4 | 1 | - | - | - | - | - | - |
| Anaesthesia | 4 | - | 1 | 3 | - | - | - | - | - | - |
| Hypoaesthesia oral | 3 | - | 2 | - | - | 1 | - | - | - | - |
| Formication | 2 | 1 | 1 | - | - | - | - | - | - | - |
| Paraesthesia oral | 2 | 1 | 1 | - | - | - | - | - | - | - |
| Burning feet syndrome | 1 | - | 1 | - | - | - | - | - | - | - |
| Hemianaesthesia | 1 | - | - | 1 | - | - | - | - | - | - |
| Hemiparaesthesia | 1 | - | - | 1 | - | - | - | - | - | - |
| Skin burning sensation | 1 | - | 1 | - | - | - | - | - | - | - |
| **Nervous system disorders NEC** | **246 (3.9)** | **12 (2)** | **123 (4.9)** | **66 (3.2)** | **1 (5.6)** | **9 (2.6)** | **-** | **14 (7.8)** | **20 (3.3)** | **1 (2.1)** |
| Metastases to centralnervous system | 86 | 5 | 47 | 10 | - | 1 | - | 13 | 9 | 1 |
| Nervous system disorder | 63 | 4 | 27 | 25 | 1 | 2 | - | - | 4 | - |
| Central nervous system lesion | 27 | 1 | 15 | 7 | - | 2 | - | 1 | 1 | - |
| Neurotoxicity | 22 | - | 10 | 8 | - | 2 | - | - | 2 | - |
| Cerebral disorder | 19 | 1 | 6 | 7 | - | 2 | - | - | 3 | - |
| Metastases to meninges | 11 | - | 10 | - | - | - | - | - | 1 | - |
| Meningeal disorder | 6 | - | 3 | 3 | - | - | - | - | - | - |
| Paraneoplastic neurological syndrome | 6 | - | 2 | 4 | - | - | - | - | - | - |
| Brain stemsyndrome | 2 | - | 2 | - | - | - | - | - | - | - |
| Central nervous system lupus | 1 | - | - | 1 | - | - | - | - | - | - |
| Central nervous system necrosis | 1 | - | 1 | - | - | - | - | - | - | - |
| Nerve injury | 1 | 1 | - | - | - | - | - | - | - | - |
| Neurodegenerative disorder | 1 | - | - | 1 | - | - | - | - | - | - |
| **Sensory abnormalities NEC** | **178 (2.8)** | **11 (1.8)** | **69 (2.7)** | **65 (3.1)** | **-** | **13 (3.8)** | **2 (5)** | **6 (3.3)** | **12 (2)** | **-** |
| Dysgeusia | 72 | 2 | 32 | 31 | - | 4 | - | 1 | 2 | - |
| Taste disorder | 25 | - | 5 | 12 | - | 1 | - | 1 | 6 | - |
| Neuralgia | 22 | 3 | 8 | 4 | - | 3 | - | 3 | 1 | - |
| Ageusia | 21 | 2 | 9 | 7 | - | 2 | - | - | 1 | - |
| Sensory disturbance | 12 | 2 | 4 | 3 | - | 2 | 1 | - |  | - |
| Sensory loss | 9 | 1 | 5 | 2 | - | - | - | - | 1 | - |
| Restless legs syndrome | 7 | - | 4 | - | - | 1 | - | 1 | 1 | - |
| Amputation stump pain | 4 | - | - | 4 | - | - | - | - | - | - |
| Hypogeusia | 2 | 1 | 1 | - | - | - | - | - | - | - |
| Aura | 1 | - | - | 1 | - | - | - | - | - | - |
| Decreased vibratory sense | 1 | - | 1 | - | - | - | - | - | - | - |
| Phantom limbsyndrome | 1 | - | - | 1 | - | - | - | - | - | - |
| Phantom pain | 1 | - | - | - | - | - | 1 | - | - | - |
| **Coordination and balance disturbances** | **139 (2.2)** | **15 (2.5)** | **58 (2.3)** | **46 (2.2)** | **-** | **4 (1.2)** | **1 (2.5)** | **6 (3.3)** | **8 (1.3)** | **1 (2.1)** |
| Balance disorder | 47 | 5 | 20 | 15 | - | 1 | - | 2 | 3 | 1 |
| Ataxia | 30 | 4 | 13 | 8 | - | 1 | 1 | 1 | 2 | - |
| Dysstasia | 27 | 3 | 7 | 14 | - | 1 | - | - | 2 | - |
| Cerebellar syndrome | 13 | 1 | 6 | 3 | - | - | - | 2 | 1 | - |
| Coordination abnormal | 10 | - | 7 | 2 | - | - | - | 1 | - | - |
| Cerebellar ataxia | 6 | - | 1 | 4 | - | 1 | - | - | - | - |
| Nystagmus | 5 | 2 | 3 | - | - | - | - | - | - | - |
| Dysmetria | 1 |  | 1 | - | - | - | - | - | - | - |
| **Coma states** | **86 (1.4)** | **10 (1.7)** | **39 (1.5)** | **21 (1)** | **-** | **4 (1.2)** | **-** | **2 (1.1)** | **9 (1.5)** | **1 (2.1)** |
| Coma | 57 | 8 | 22 | 16 | - | 4 | - | - | 7 | - |
| Diabetic ketoacidotic hyperglycaemic coma | 9 | - | 5 | 2 | - | - | - | 2 | - | - |
| Diabetic hyperglycaemic coma | 4 | 1 | 3 | - | - | - | - | - | - | - |
| Coma hepatic | 3 | 1 | 2 | - | - | - | - | - | - | - |
| Diabetic coma | 3 | - | 1 | 1 | - | - | - | - | 1 | - |
| Diabetic hyperosmolar coma | 3 | - | 2 | - | - | - | - | - | - | 1 |
| Hypoglycaemic coma | 3 | - | 1 | 1 | - | - | - | - | 1 | - |
| Coma acidotic | 2 | - | 1 | 1 | - | - | - | - | - | - |
| Hypercapnic coma | 2 | - | 2 | - | - | - | - | - | - | - |
| **Speech articulation and rhythm disturbances** | **64 (1)** | **3 (0.5)** | **28 (1.1)** | **22 (1.1)** | **1 (5.6)** | **-** | **-** | **4 (2.2)** | **6 (1)** | **-** |
| Dysarthria | 43 | 2 | 14 | 20 | 1 | - | - | 1 | 5 | - |
| Dysphonia | 14 | - | 13 | - | - | - | - | - | 1 | - |
| Dyslalia | 7 | 1 | 1 | 2 | - | - | - | 3 | - | - |
| **Speech and language abnormalities** | **57 (0.9)** | **3 (0.5)** | **28 (1.1)** | **19 (0.9)** | **1 (5.6)** | **3 (0.9)** | **-** | **1 (0.6)** | **2 (0.3)** | **-** |
| Speech disorder | 51 | 3 | 26 | 17 | - | 3 | - | 1 | 1 | - |
| Incoherent | 3 | - | - | 2 | - | - | - | - | 1 | - |
| Language disorder | 3 | - | 2 | - | 1 | - | - | - | - | - |
| **Cortical dysfunction NEC** | **55 (0.9)** | **3 (0.5)** | **23 (0.9)** | **19 (0.9)** | **1 (5.6)** | **1 (0.3)** | **1 (2.5)** | **-** | **6 (1)** | **1 (2.1)** |
| Aphasia | 48 | 2 | 22 | 17 | 1 | 1 | 1 | - | 3 | 1 |
| Acalculia | 1 | - | - | - | - | - | - | - | 1 | - |
| Agnosia | 1 | - | - | - | - | - | - | - | 1 | - |
| Alexia | 1 | - | - | - | - | - | - | - | 1 | - |
| Apraxia | 1 | - | 1 | - | - | - | - | - | - | - |
| Dyspraxia | 1 | - | - | 1 | - | - | - | - | - | - |
| Prosopagnosia | 1 | - | - | 1 | - | - | - | - | - | - |
| Psychomotor disadaptation syndrome | 1 | 1 | - | - | - | - | - | - | - | - |
| **Vertigos NEC** | **20 (0.3)** | **3 (0.5)** | **13 (0.5)** | **1 (0.05)** | **-** | **-** |  | **-** | **4 (0.7)** | **-** |
| Vertigo | 19 | 2 | 13 | - | - | - | - | - | 4 | - |
| Vestibular neuronitis | 1 | - | - | 1 | - | - | - | - | - | - |
| **Abnormal reflexes** | **12 (0.2)** | **1 (0.2)** | **5 (0.2)** | **4 (0.2)** | **-** | **1 (0.3)** | **1** | **-** | **-** | **-** |
| Areflexia | 7 | - | 2 | 4 | - |  | 1 | - | - | - |
| Hyporeflexia | 3 | 1 | 1 | - | - | 1 | - | - | - | - |
| Hyperreflexia | 2 | - | 2 | - | - | - | - | - | - | - |
| ***Total*** | ***2066 (33.1)*** | ***144 (23.9)*** | ***853 (33.8)*** | ***683 (33)*** | ***6 (33.3)*** | ***114 (33.2)*** | ***21 (52.5)*** | ***76 (42.2)*** | ***163 (26.8)*** | ***9 (18.7)*** |
